# Enabling Aboriginal dental assistants to apply fluoride varnish for school children in communities with a high Aboriginal population in New South Wales, Australia: a study protocol for a feasibility study

**DOI:** 10.1186/s40814-019-0399-4

**Published:** 2019-01-22

**Authors:** Yvonne Dimitropoulos, Anthony Blinkhorn, Michelle Irving, John Skinner, Steven Naoum, Alexander Holden, Angela Masoe, Boe Rambaldini, Vita Christie, Heiko Spallek, Kylie Gwynne

**Affiliations:** 1 Poche Centre for Indigenous Health, The University of Sydney, Room 224 Edward Ford Building, Sydney, 2006 Australia; 2 Faculty of Medicine and Health, The University of Sydney School of Dentistry, 1 Mons Road, Westmead, NSW 2145 Australia; 3 NSW Centre for Oral Health Strategy, 73 Miller Street, North Sydney, NSW 2060 Australia

**Keywords:** Fluorides, Topical, Dental assistants, Oral health, Aboriginal

## Abstract

**Background:**

Australian Aboriginal children experience high levels of dental caries (tooth decay) and are less likely to access preventive dental health services. High-strength fluoride varnish has been shown to reduce the incidence of dental caries and is commonly used in community-based preventive dental health service programs. In New South Wales, Australia, the application of fluoride varnish is restricted to dental and medical professionals. This is problematic in communities with a high Aboriginal population and limited access to oral health services, contributing to the increased risk of developing dental caries in Aboriginal children. Dental assistants are essential members of the oral health team; however, they do not have a defined scope of practice in Australia. Other countries have created formal scopes of practice for dental assistants to include the application of fluoride varnish. This protocol presents a pathway for qualified Aboriginal dental assistants to undertake additional training to legally apply fluoride varnish in New South Wales. The primary objective of this study will be to evaluate the feasibility and acceptability of utilising Aboriginal dental assistants to apply fluoride varnish to Aboriginal children in a school setting at regular 3-month intervals.

**Methods:**

Six schools across New South Wales (NSW) that enrol at least 12% Aboriginal children will be invited to participate in the 12-month study. Aboriginal children aged 5–12 years enrolled in these schools will be enrolled in the study. Six Aboriginal dental assistants will undertake training to apply fluoride varnish. Fluoride varnish (Duraphat) will be applied at 3-month intervals by the dental assistants to the teeth using a small brush. An evaluation will be undertaken to determine the feasibility and cost-effectiveness of this innovative approach. This study protocol has been approved by the NSW Aboriginal Health and Medical Research Council and the NSW State Education Research Application Process.

**Discussion:**

A qualified Aboriginal dental assistant workforce in NSW (or Australia) legally approved to apply fluoride varnish may increase the sustainability and scalability of fluoride varnish programs and improve the oral health of Aboriginal children in Australia.

**Trial registration:**

ISRCTN26746753.

**Electronic supplementary material:**

The online version of this article (10.1186/s40814-019-0399-4) contains supplementary material, which is available to authorized users.

## Background

Dental caries (tooth decay) is a widespread chronic disease among Australian children which is largely preventable [1, 2]. Australian Aboriginal children experience higher levels of dental caries than non-Aboriginal children. In New South Wales (NSW) for example, Aboriginal children experience 2.64 teeth on average affected by dental caries, compared to 1.54 teeth for non-Aboriginal children [3]. Untreated dental caries can cause considerable pain, impacting on a child’s school attendance, ability to chew or speak normally and overall quality of life [4].

Varnishes which contain higher levels of fluoride were developed in the 1960s to prevent dental caries [5]. They are safe [6] and widely used internationally in community-based preventive dental health service programs [7]. A meta-analysis of clinical trials undertaken by the Cochrane review aimed to assess the caries preventive effect of fluoride varnish concluded that fluoride varnish applied to the teeth two to four times per year is associated with a substantial reduction in caries development [7]. This included a 43% reduction in decayed, missing and filled tooth surfaces in the permanent dentition and 37% reduction in the primary dentition.

Under current Australian legislation (with exception to the Northern Territory), only medical and dental professionals can apply fluoride varnish [8]. Due to the uneven supply of oral health professionals in rural areas [9, 10], there is limited access to evidence-based preventive dental health services in rural and remote communities with high Aboriginal populations. This contributes to the complex matrix of risk factors for the development of dental caries in Aboriginal children. In NSW, a pathway exists to allow non-medical or dental professionals to apply fluoride varnish following approval from the Chief Health Officer [11].

In the Northern Territory, non-dental professionals including Aboriginal Health Workers, Aboriginal Health Practitioners and school or child health nurses are legally able to apply fluoride varnish, following mandatory training [12]. This initiative assists in improving access to preventive services for Aboriginal children. Improving the oral health of priority populations including Aboriginal people is documented in Australia’s National Oral Health Plan. The plan also strongly supports the expansion of fluoride varnish programs and seeks greater uniformity of Australian legislation to support fluoride varnish programs which may improve oral health of priority populations [13]. Furthermore, the NSW State Government has developed a set of guidelines to increase access to fluoride for target populations in NSW. These include the implementation of community fluoride varnish programs [14].

Dental assistants are essential members of the oral health team. Whilst they are not regulated by the Dental Board of Australia and as such do not have a defined scope of practice, in NSW, Victoria and South Australia, dental assistants are included in legislative frameworks that impose standards on non-registered health practitioners who are involved in the provision of health services [15]. In Australia, vocational training is available for dental assistants to become qualified. This training is administered and delivered through a Registered Training Organisation with an approved curriculum that includes cross-infection control and dental anatomy, both of which are relevant to the application of fluoride varnish.

Other countries have created formal scopes of practice for dental assistants to include the application of fluoride varnish [16, 17]. In the UK, for example, the General Dental Council allows trained and competent dental nurses to apply fluoride varnish direct in community-based fluoride varnish programs and under the direction of a dentist in practices. Eighty percent of these dental nurses expressed they were confident applying fluoride varnish [17]. In Scotland, as part of the Childsmile program, Extended Duties Dental Nurses (EDDNs) provide fluoride varnish applications to preschool children. The EDDNs showed professionalism when working with children, and no incidents had been reported [16].

A small-scale study in Central Northern NSW, Australia, used oral health therapists to deliver 3-monthly applications of fluoride varnish to Aboriginal school children [18]. The study provided at least three fluoride varnish applications for Aboriginal children over a 12-month period and noted that such an approach could be modified to use dental assistants to increase the sustainability of the program [18]. The proposed pilot project will enable dental assistants to apply fluoride varnish as an alternative to oral health professionals and may increase the sustainability and scalability and reduce the costs of fluoride varnish programs. This approach may also increase access to evidence-based preventive dentistry for Aboriginal children living in communities with limited dental services.

This study protocol presents a pathway for qualified Aboriginal dental assistants to undertake additional training to legally apply fluoride varnish in NSW. The primary objective of this 12-month, single group study across seven schools will be to test the feasibility and acceptability of Aboriginal dental assistants applying fluoride varnish in a school setting for children at risk of dental caries. Secondary objectives of this study are as follows:

1. To determine the feasibility of utilising Aboriginal dental assistants to apply fluoride varnish at regular 3-month intervals
2. To determine whether this model can provide Aboriginal children with at least three applications of fluoride varnish over the 12-month study period
3. To explore the confidence of dental assistants applying fluoride varnish
4. To explore the acceptability of using dental assistants to deliver fluoride varnish in the school setting
5. To determine the cost-effectiveness of utilising dental assistants to provide four fluoride varnish applications in the school setting over the 12-month study period compared to oral health therapists

## Methods

### Setting

Aboriginal Community Controlled Health Services are health care services that are operated by the local Aboriginal community. They deliver holistic, culturally competent health care to the local Aboriginal community and liaise with local organisations such as schools on issues affecting the health and well-being of Aboriginal people [19]. Six Aboriginal Community Controlled Health Services in communities across NSW with a high Aboriginal population will be invited to participate in the study. Each Aboriginal Community Controlled Health Service will be required to nominate one Aboriginal dental assistant and a local school/s to participate. Each Aboriginal Community Controlled Health Service will be given a set of criteria to select a local school to participate in the fluoride varnish program. The criteria will include (i) school enrols at least 12% Aboriginal children, (ii) children at the school experience high levels of untreated dental caries, (iii) community water is non-fluoridated or children consume high levels of sugar-sweetened beverages, and (iv) school does not receive another fluoride varnish program. A 12% minimum enrolment of Aboriginal children was selected as this is four times the average population proportion of Aboriginal people in Australia [20]. Once a local school meets the selection criteria, the Aboriginal Community Controlled Health Service will engage with the school principal to seek consent to implement the program at the school.

### Study design

Each Aboriginal Community Controlled Health Service will nominate one Aboriginal dental assistant to participate in the program, who has already completed a Certificate III in Dental Assisting. Each dental assistant will need to successfully complete additional training to be able to apply the fluoride varnish. The further training will be administered through NSW TAFE (HLTOHC006 Apply Fluoride Varnish). Each dental assistant will also receive training on the fluoride varnish application protocol developed specifically for this study (Additional file 1). Each dental assistant will be required to review the Health Care Complaints Commission’s Code of Conduct for unregistered health professionals, which applies to non-registered health practitioners providing healthcare services in NSW [21].

The six dental assistants will need to provide two references, a ‘Working With Children Check’ from NSW and a National Police Check from the Australian Federal Police. References and relevant checks will be sighted by a fluoride varnish pilot project supervisor. Upon completion of this vetting process, the names of the six dental assistants will be forward to the Chief Health Officer for NSW for approval to legally apply fluoride varnish through an exemption under the *NSW Poisons and Therapeutic Goods Regulations Act 2008*.

### Sample size

This study aims to recruit 500 Aboriginal children which constitute approximately 1% of all Aboriginal children enrolled in public schools in NSW [22]. Furthermore, six Aboriginal Community Controlled Health Services across NSW will be invited to participate in the study representing 15% of all Aboriginal Community Controlled Health Services across NSW.

### Recruitment of participants

Each school must also agree to take an active role in managing the consent process to enrol children into the study. The school will be required to engage an Aboriginal Education Officer to liaise with Aboriginal families to explain the consent form and enrol children into the study. This approach will allow Aboriginal families to ask questions about the program and provide informed consent for their child to participate in the study and aims to increase student participation and retention.

### Inclusion and exclusion criteria

Aboriginal children aged 5–12 years will be eligible to participate in the study. Inclusion of non-Aboriginal children will be allowed at the school’s request. Parents and/or guardians will be asked to complete a medical history questionnaire along with the consent form to ascertain each child’s asthma and allergy status. Children with uncontrolled asthma and a history of allergy to resins will be excluded [23].

### Description of intervention

Four days over the 12-month study period will be scheduled with the school for each dental assistant to apply the fluoride varnish to all eligible children. These days are known as ‘fluoride varnish days’. One ‘fluoride varnish day’ will take place each school term, and days must be at least 3 months apart. One further day will be scheduled 1 week after each ‘fluoride varnish day’ to follow up children who were absent. Those absent on the follow-up date will not receive a fluoride varnish application in that 3-month period.

Prior to the first fluoride varnish application by the dental assistant, a caries risk assessment developed specifically for this study will be completed by a supervising oral health therapist for each child with a valid consent form and no contraindicating medical history [Additional file 2]. Children who present with untreated dental caries will be referred to the local Aboriginal Community Controlled Health Service for dental treatment. Children identified as ‘at risk’ of developing dental caries will be enrolled into the study.

**Fig. 1:**
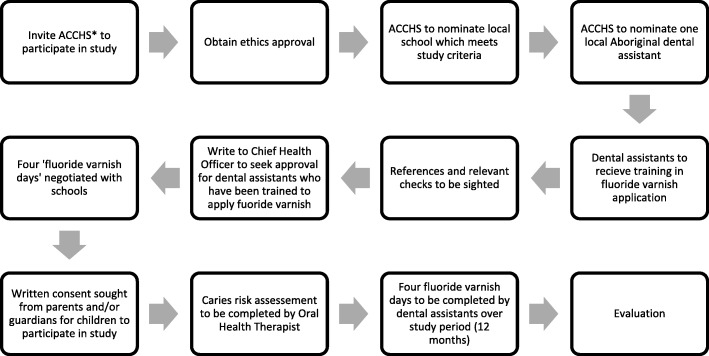
Process flow of the proposed study

Duraphat fluoride varnish will be used as part of this study. Each fluoride varnish application will follow the protocol developed specifically for this study [Additional file 1]. Fluoride varnish applications will take place in a classroom with the child seated on a standard school chair. Teeth will be examined for visible plaque; if a substantial amount of plaque is present, children will be asked to clean their teeth with a toothbrush but no toothpaste. Teeth will be dried using one piece of gauze, and a thin film of 0.4 ml of Duraphat fluoride varnish [23] will be applied to the interproximal (in-between) and occlusal (chewing) surfaces of the teeth using a small brush. These surfaces are most susceptible to developing dental caries [24]. Fluoride varnish will be dispensed onto a 0.4-ml dispensing pad provided by the manufacturer, to ensure the correct amount is dispensed. Any residual fluoride varnish on the soft tissues of the mouth will be wiped off with a second piece of gauze. Each child will be given verbal post-operative instructions, as well as being issued with a sticker. Instructions will include abstention from food for 30 min and to resume toothbrushing normally in the evening. The sticker will note the time food consumption can resume. An oral health therapist will provide on-site supervision for the first fluoride varnish application and remote supervision for the remainder of the study period. In the unlikely event of an adverse reaction to the fluoride varnish, the child will discontinue enrolment in the study. Adverse reactions will be recorded in the ‘Fluoride varnish day’ summary sheet (Additional file 3). Figure 1 demonstrates the process flow of the proposed study.

### Data analysis

A process, impact and outcome evaluation will be undertaken based on the Quality Implementation Framework [25]. The process evaluation will include readiness and pre-training questionnaires for the dental assistants and supervising oral health therapists to determine the confidence of dental assistants applying fluoride varnish in the school setting. Impact measures will include the number of Aboriginal dental assistants and supervising oral health therapists participating in the study, satisfaction of key stakeholders to determine if schools are an appropriate setting for a fluoride varnish program and the acceptability of using dental assistants to deliver the program. Each fluoride varnish application will be recorded using a study-specific item number in the child’s confidential record stored in the local ACCHS’ dental software system. At the completion of the study, the item numbers related to the study and the demographics of each child will be extracted from each ACCHS’ dental software system. Descriptive analysis of fluoride varnish applications for all children included in the study will be performed using SAS Statistical software. The proportion of children who received one, two, three or four fluoride varnish applications over the study period will be presented with 95% CIs. This will determine whether this program can provide Aboriginal children at least three fluoride varnish applications over the 12-month study period. A cost analysis will also be undertaken to determine the overall cost-effectiveness of using dental assistants to apply fluoride varnish four times per year to school children compared to oral health therapists.

## Discussion

This protocol describes the methodology of a proposed study which aims to determine the feasibility and acceptability of using Aboriginal dental assistants to apply fluoride varnish to Aboriginal school children four times per year in communities with a high Aboriginal population. The proposed study has been developed in response to the long-standing priority in Australia, and specifically NSW, to develop evidence-based, culturally competent and sustainable public oral health programs to improve the oral health of Australian Aboriginal children [13, 26, 27]. This study provides a process for systematic and scalable application of fluoride varnish to vulnerable populations.

The proposed pilot project will take place in communities with a high Aboriginal population however could be adapted for communities where there are high rates of dental caries, limited access to oral health services, non-fluoridated water supply or high consumption of sugar-sweetened beverages in children.

Oral health promotion aimed at improving the oral health of Aboriginal people is more likely to be effective if the programs have been co-designed with local Aboriginal communities, meeting their specific needs and achieving community ownership [28–30]. The protocol for this study was developed in collaboration with six Aboriginal communities across NSW. Each community was also invited to nominate local Aboriginal investigators to participate in the design and future implementation of the study.

This pilot project will enable Aboriginal Community Controlled Health Services to lead development and implementation of the study in their community. The health services are given the opportunity to identify a local school/s they believe will benefit from the program and engage closely with schools to implement and monitor the program. This is an important component of the design of this study and assists in achieving community ownership and may increase adoption of the program in schools. Furthermore, this study will take place in schools and a local Aboriginal dental assistant will apply the fluoride varnish, promoting a culturally competent environment for Aboriginal children to access a preventive oral health service. Following the completion of the study, the results will be reported back to each local community, allowing the community including the school and Aboriginal Community Controlled Health Services to provide feedback on the program.

An exemption has been sought from the Chief Health Officer for NSW under the *Poisons and Therapeutic Goods Regulations Act 2008*, to legally enable dental assistants to apply fluoride varnish in this pilot study. This pilot will be evaluated to determine the feasibility of utilising dental assistants to apply fluoride varnish at 3-monthly intervals in the school setting. The results of this pilot will be reported to the Chief Health Officer for NSW to propose the development of a scaling study to include an additional ten schools to determine if this approach is effective at scale. The development of a state or national model which would legally enable dental assistants to apply fluoride varnish four times per year in schools may increase the sustainability and scalability of fluoride varnish programs and improve the oral health of Aboriginal children in Australia.

This study protocol presents a process for Aboriginal dental assistants to apply fluoride varnish four times per year to Aboriginal school children in communities with a high Aboriginal population. The proposed pilot project aims to determine the efficacy of this approach and establish new, sustainable and scalable ways to enhance public oral health. Dental disease is largely preventable, and low-cost efficacious programs are critical to reducing the burden of disease in vulnerable populations.

## Additional files

Additional file 1: Caries risk assessment. (DOCX 80 kb)

Additional file 2: Fluoride varnish day protocol. (DOCX 14 kb)

Additional file 3: Fluoride varnish day summary sheet. (PDF 130 kb)

## Abbreviations

AH&MRC: Aboriginal Health and Medical Research Council (NSW)
ICMJE: International Committee of Medical Journal Editors
NSW: New South Wales
