# Supplementary material for: Enabling Aboriginal dental assistants to apply fluoride varnish for school children in communities with a high Aboriginal population in New South Wales, Australia: a study protocol for a feasibility study
Source: Pilot Feasibility Stud. 2019 Jan 22;5:15. doi: 10.1186/s40814-019-0399-4 (PMC6341707; doi:10.1186/s40814-019-0399-4)
Supplement: Supplementary file 1 — Caries risk assessment. (DOCX 80 kb) [file 40814_2019_399_MOESM1_ESM.docx]

Additional File 1: Caries Risk assessment

Patient name: __________________________ Date: ________________________________

1. Fluoride tooth paste exposure

Did the patient FAIL to brush their teeth with a fluoride toothpaste last night?

Y / N

1. Diet assessment
   1. Did the patient drink ANY of the following yesterday: fruit juice; cordial; fizzy drinks; energy drinks?

Y / N

- 1. Did the patient eat ANY of the following yesterday: biscuits; cakes; chocolates; lollies?

Y / N

1. Plaque assessment

Criteria for plaque assessment (Silness & Loe, 1964)

3 = thick plaque is visible along gingival margin (no need to probe)

2 = plaque is visible along gingival margin, with or without air drying (no need to probe)

1 = plaque is not visible but can be wiped off with an explorer

0 = plaque is not visible not cannot be wiped off with an explorer


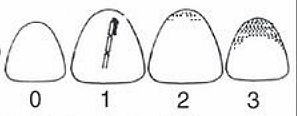

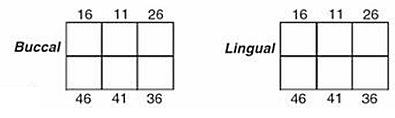


Notes:

1. If an index tooth is missing, score the nearest tooth in that sextant. If there are no teeth in the sextant, enter X
2. If plaque thickness varies along the gingival margin, score according to the worst situation

Was a score of 3 recorded for any site?

Y/N

1. Caries diagnosis and history
   1. Are there any sites demonstrating dentine shadowing?

Y / N

- 1. Are there any sites demonstrating cavitation?

Y / N

- 1. Are there any sites exhibiting a restoration?

Y / N

Caries risk categorisation and study inclusion:

If any of the above questions were answered Yes – the patient is at risk of developing caries and should be included in the study following gingival assessment.

Gingival assessment: Absence of ulcerative gingivitis or stomatitis? Y / N

Child eligible for fluoride varnish application: Y / N

Signed: _________________________
