# Supplementary material for: Enabling Aboriginal dental assistants to apply fluoride varnish for school children in communities with a high Aboriginal population in New South Wales, Australia: a study protocol for a feasibility study
Source: Pilot Feasibility Stud. 2019 Jan 22;5:15. doi: 10.1186/s40814-019-0399-4 (PMC6341707; doi:10.1186/s40814-019-0399-4)
Supplement: Supplementary file 2 — Fluoride varnish day protocol. (DOCX 14 kb) [file 40814_2019_399_MOESM2_ESM.docx]

Additional File 2: Fluoride varnish day protocol

Materials for each child

• PPE

• 1 x microbrush applicator

• 1 x medium size patient bib

• 0.4ml dispensed onto fluoride varnish dispensed onto pad

• 2 x gauze

• Toothbrush

• Plastic cup

• Disposable mirror

Protocol for first fluoride varnish day (Oral Health Therapists)

1. Confirm child’s name; asthma history; and resin allergy history on consent form. Asthma must be controlled; no known allergy to resin for a child to be eligible to receive varnish application.
2. Search for existing Titanium file or create new Titanium file if consent recently returned to school. If a child has received a fluoride varnish application within 3 months on their Titanium file, exclude child from today’s fluoride varnish application but proceed with risk assessment for future fluoride varnish days.
3. Complete risk assessment for each child and record ‘013’ in Titanium. Patient must be at risk of caries and no signs of ulcerative gingivitis or stomatitis to be eligible for the study. Record clinical notes and click ‘charge’ to save
4. Handover to Dental Assistant for fluoride varnish application.

Protocol for each fluoride varnish application (Dental Assistants)

1. Place a medium sized bib on table and prepare materials
2. Confirm child’s name; asthma history; and resin allergy history on consent form. Asthma must be controlled; no known allergy to resin for a child to be eligible to receive varnish application.
3. Search for existing Titanium file or create new Titanium file if consent recently returned to school. If a child has received a fluoride varnish application within 3 months on their Titanium file, exclude child from today’s fluoride varnish application. Child may be eligible for next fluoride varnish day.
4. Explain application of fluoride varnish to child
5. Perform hand hygiene and put on personal protective equipment
6. Examine teeth for visible plaque. If an abundance of plaque is present, ask child to brush their teeth with tooth brush but not using toothpaste
7. Ask child to rinse mouth out with water if necessary
8. Dry teeth using 1 piece of gauze
9. Apply a thin film of fluoride varnish to occlusal and interproximal surfaces of posterior teeth and buccal surfaces of maxillary anterior teeth
10. Wipe any residual fluoride varnish on soft tissue with second piece of gauze
11. Give child post-operative instructions (see below). Child able to return to class
12. Place contaminated items in bin and don gloves
13. Put on new gloves and wipe down contaminated work surface
14. Don personal protective equipment and wash hands using alcohol rub
15. Record item number ‘121’ in patient’s Titanium file and that fluoride varnish was applied in clinical notes. Click ‘charge’ to save
16. Record child has received fluoride varnish on class role.

Post-operative instructions

• No food or drink for 30 minutes. Issue child with sticker and write time that eating can resume

• Tooth brushing can resume normally tonight
