# Supplementary material for: Enabling Aboriginal dental assistants to apply fluoride varnish for school children in communities with a high Aboriginal population in New South Wales, Australia: a study protocol for a feasibility study
Source: Pilot Feasibility Stud. 2019 Jan 22;5:15. doi: 10.1186/s40814-019-0399-4 (PMC6341707; doi:10.1186/s40814-019-0399-4)
Supplement: Supplementary file 3 — Fluoride varnish day summary sheet. (PDF 130 kb) [file 40814_2019_399_MOESM3_ESM.pdf]

Poche Centre for Indigenous Health - Fluoride varnish program
Data collection summary sheet

Instructions:

1. This data collection summary sheet is to be completed by the Dental Assistant at the end of each fluoride varnish day and follow up fluoride varnish day
2. Send completed data collection summary sheet PLUS Titanium Service Profile report to Dr Kylie Gwynne (contact details below) at the end of each fluoride varnish day and follow up fluoride varnish day.

**Fluoride vanish day – complete questions 1 + 2**<br>**Follow up fluoride varnish day – complete question 3**

Contact:

Send completed data collection summary sheet PLUS Titanium Service Profile Report:

Dr Kylie Gwynne
Chief Investigator
Kylie.gwynne@sydney.edu.au

If you have any trouble completing this form:

Yvonne Dimitropoulos
Oral health promotion coordinator
02 9114 0829
04 11 702 930

School details:

School: ____ Date: ________________________

School fluoride varnish day: (Please circle) 1 2 3 4

Aboriginal Medical Service: ____

Supervising Oral Health Therapist: ____

Dental Assistant: ____

Fluoride varnish batch number/s: ______________________________

Poche Centre for Indigenous Health - Fluoride varnish program
Data collection summary sheet

Questions:

1) Support from school on fluoride varnish day:

Item	Yes / No
School provided a room for the program	
School provided 1 teacher or Aboriginal Education Officer to assist the program	

How could this be improved for the next fluoride varnish day? ________________________
____

2) Fluoride varnish day summary:

	Item	Total number
1	Number of children at the school that are eligible for the program	
2	Number of children with signed consent returned in the time frame to participate in the program	
3	Number of children excluded due to contra-indicating medical history	
4	Number of children excluded following risk assessment by OHT	
5	Number of children eligible for fluoride varnish application (Item 2 – [Items 3+4])	
6	Number of children who received fluoride varnish application today	
7	Number of children who did not receive fluoride varnish application today due to being absent (to be followed up on follow up fluoride varnish day in 1 week)	
8	Number of children who did not receive fluoride varnish application today due to refusing application (to be followed up on follow up fluoride varnish day in 1 week)	
9	Number of children to follow up on follow up fluoride varnish day in 1 week	
10	Number of children who experienced an adverse reaction or any type of reaction following fluoride varnish application	

3) Follow up fluoride vanish day summary

	Item	Total number
1	Number of children required for follow up	
2	Number of children who received fluoride varnish application today	
3	Number of children who did not receive fluoride varnish application today due to being absent (do not receive fluoride varnish application this term)	
4	Number of children who did not receive fluoride varnish application today due to refusing application (do not receive fluoride varnish application this term)	
5	Number of children who did not receive fluoride varnish application this term (Item 3 + 4)	
